# Neuroimmune Interaction: A Widespread Mutual Regulation and the Weapons for Barrier Organs

**DOI:** 10.3389/fcell.2022.906755

**Published:** 2022-05-11

**Authors:** Yan Zhu, Shixin Duan, Mei Wang, Zhili Deng, Ji Li

**Affiliations:** ^1^ Department of Dermatology, Xiangya Hospital, Central South University, Changsha, China; ^2^ Hunan Key Laboratory of Aging Biology, Xiangya Hospital, Central South University, Changsha, China; ^3^ National Clinical Research Center for Geriatric Disorders, Xiangya Hospital, Central South University, Changsha, China

**Keywords:** neuroimmune crosstalk, nerve, immune, barrier organ, neuropeptide, neurotransmitter

## Abstract

Since the embryo, the nervous system and immune system have been interacting to regulate each other’s development and working together to resist harmful stimuli. However, oversensitive neural response and uncontrolled immune attack are major causes of various diseases, especially in barrier organs, while neural-immune interaction makes it worse. As the first defense line, the barrier organs give a guarantee to maintain homeostasis in external environment. And the dense nerve innervation and abundant immune cell population in barrier organs facilitate the neuroimmune interaction, which is the physiological basis of multiple neuroimmune-related diseases. Neuroimmune-related diseases often have complex mechanisms and require a combination of drugs, posing challenges in finding etiology and treatment. Therefore, it is of great significance to illustrate the specific mechanism and exact way of neuro-immune interaction. In this review, we first described the mutual regulation of the two principal systems and then focused on neuro-immune interaction in the barrier organs, including intestinal tract, lungs and skin, to clarify the mechanisms and provide ideas for clinical etiology exploration and treatment.

## 1 Introduction

Adapting to environmental changes and maintaining homeostasis requires the involvement of both the nervous system and immune system (Veiga-Fernandes and Pachnis, 2017; Zhang et al., 2021). The nervous system dominates tissues or organs mainly through a variety of neurotransmitters, while the immune system resists pathogens by phagocytosis and immune-active substances. According to research in recent years, the two systems can be linked together as a whole through certain agents, such as neurotransmitters, cytokines, hormones, etc (Chavan et al., 2017; Reardon et al., 2018). Neurotransmitter receptors are distributed on some immune cells, which are the foundation of neuromodulation of the immune system. And there are also cytokine receptors on nerve endings, which are the essential pathway for immunomodulation of the nervous system. The nervous system speeds up the immune response, and the immune system correspondingly helps transfer information to the nervous system. Neuro-immune interaction enriches the body’s response to the environment. However, under certain circumstances, neuro-immune interactions are the driving factors for the onset and progression of numerous diseases (Kabata and Artis, 2019; Tanaka and Okusa, 2019; Scheiblich et al., 2020).

For the past few years, there is increasing evidence about the mutual regulation of nerve and immune systems, which reveals that nerves regulate the generation, maturation, and release of immune cells (Elenkov et al., 2000), and immunity is involved in neural development (Cowan and Petri, 2018; Vainchtein et al., 2018). As the first line of defense, the gut-skin-lung barrier plays an important role in clearing harmful substances, resisting pathogen invasion, and maintaining homeostasis, and its dysfunction leads to the occurrence and progression of diseases. What’s more, there are dense nerve innervation and resident immune cell groups on the barrier, and the neuro-immune interaction occupies a more significant position in the pathogenesis of diseases. For example, neuropeptides secreted by sensory nerves trigger neurogenic inflammation and lead to allergic diseases (Sousa-Valente and Brain, 2018), while sympathetic and parasympathetic nerves produce neurotransmitters binding to different receptors on immune cells for immune regulation (Kenney and Ganta, 2014). Cytokine receptors on nerve endings collect information of immune status and transmit feedback signs.

**FIGURE 1:**
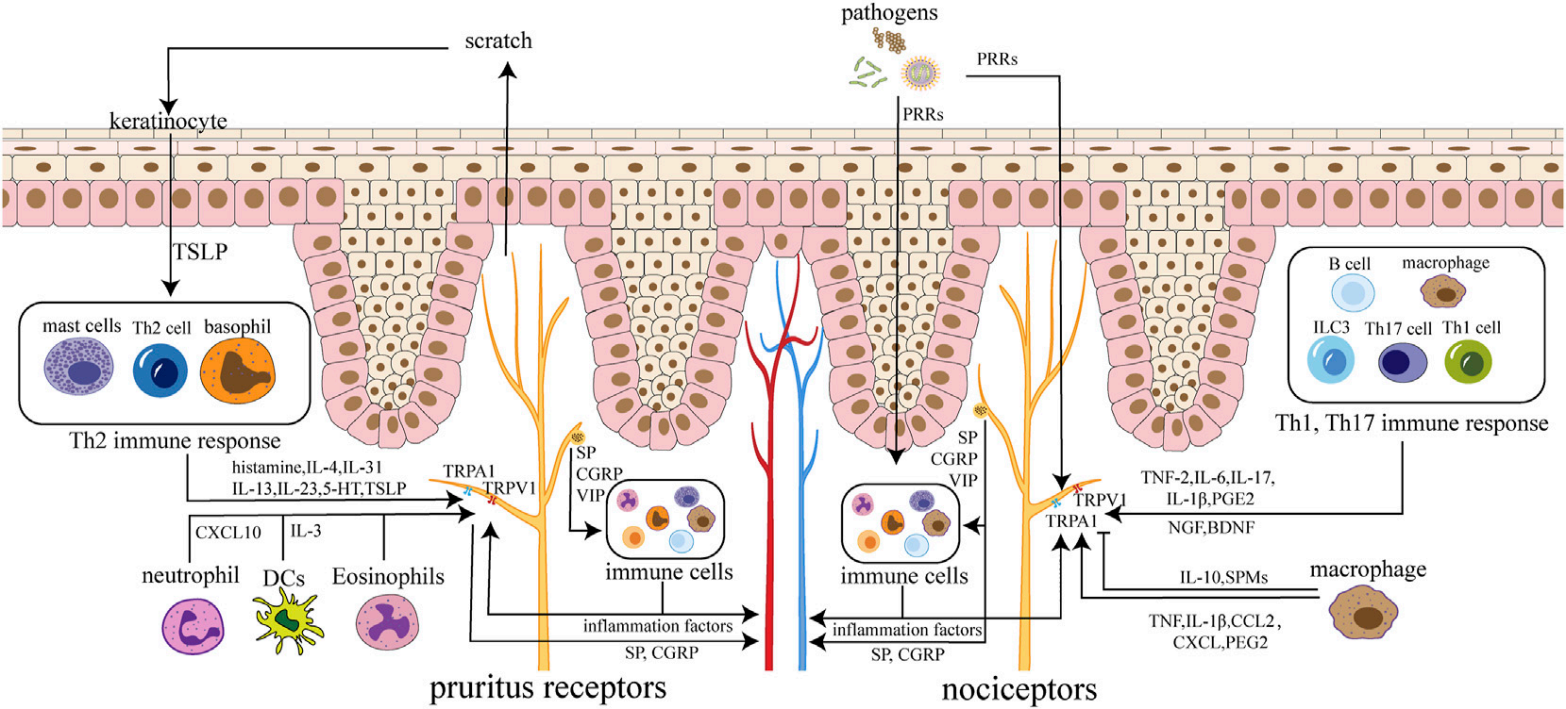
Neuroimmune circuits in skin. Immune cells activate receptors and channels on sensory neurons in the skin by secreting cytokines, chemokines, and lipids, leading to itching and pain. Nerve endings can also regulate immune cells by releasing SP, CGRP, VIP and other neuropeptides. Neuropeptides and factors can act on the skin and blood vessels, causing inflammation and edema. Pathogens can stimulate nociceptors directly or by inflammatory cytokines from immune cells. Pruritus receptors’ activation cause scratch, which leads to the destruction of keratinocytes with TSLP released, triggering amplification effect. Nociceptors are mainly involved in Th1 and Th17 immune responses, while pruritus receptors are mainly involved in Th2 immune responses.

**FIGURE 2:**
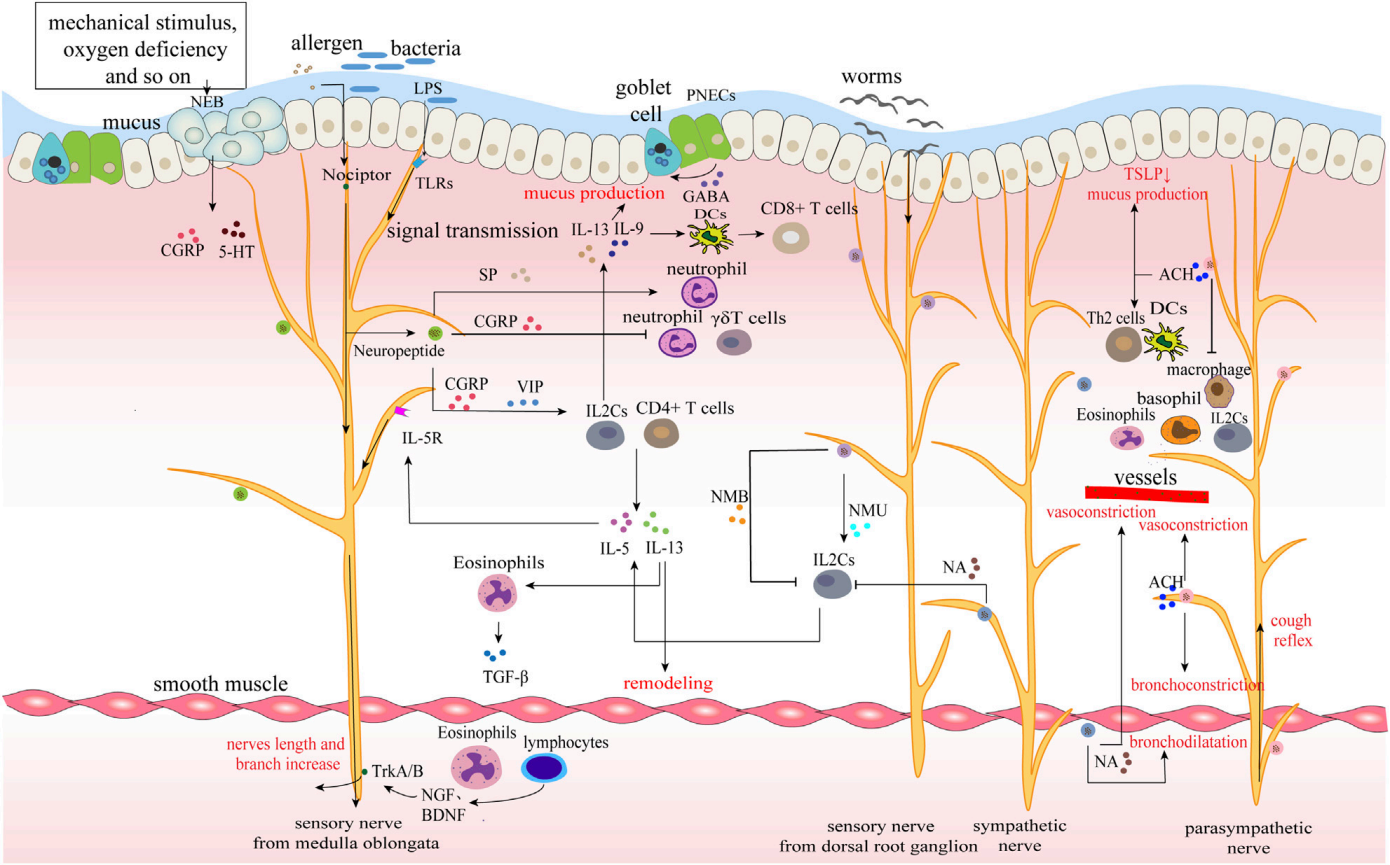
Neuroimmune circuits in lungs. The epithelium, nerves and immunity of the lung together constitute the airway barrier. Various allergens, bacteria and parasites stimulate and interact with different nerves to upload signals and regulate immunity. For example, LPS of bacteria combines with TLRs of sensory nerves to promote the release of neuropeptides, while worms act on sympathetic nerves to secrete neurotransmitters and mainly regulate ILC2s. Immune cells transmit immune signals to the nerve by releasing cytokines, forming a closed-loop of nerve and immunity. Moreover, immune cells synthesize nerve growth factor to promote nerve growth and increase nerve exposure in the airway.

**FIGURE 3:**
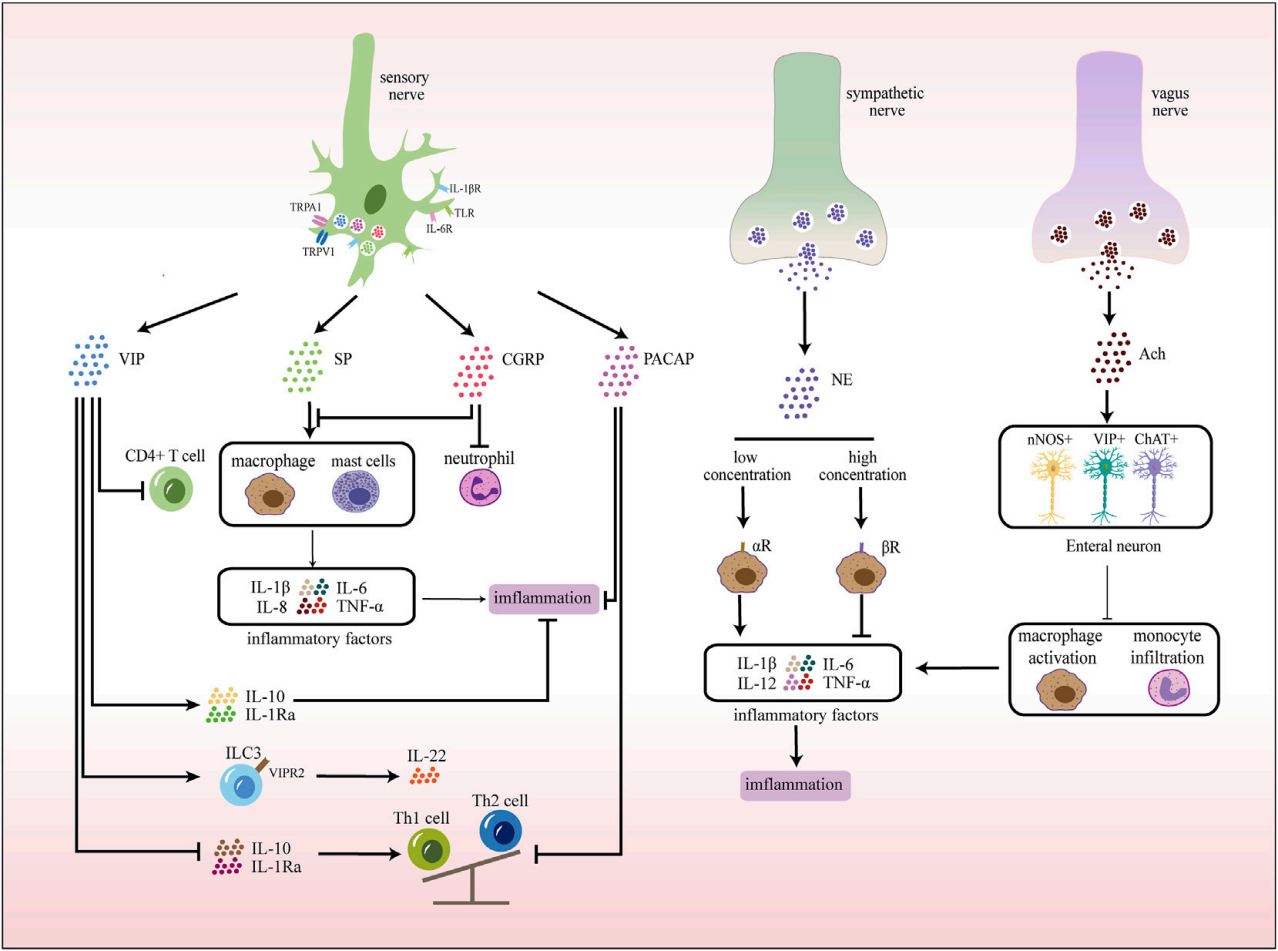
Neuroimmune communication in the intestinal tract. In the gut, sensory nerves express receptors that recognize various stimuli, pathogens, and cytokines. Activated sensory nerves release different neurotransmitters that interact with immune cells. In addition, exogenous sympathetic and vagus nerves also participate in inflammatory responses by releasing corresponding neurotransmitters.

At present, the mechanism of neuro-immune interactions remains unclear, and individualized treatment is hard to practice. Further basic research and clinical trials are needed to find out the pattern of neuroimmune-related disease and provide a more rational therapeutic regimen.

## 2 The Mutual Regulation of Nervous System and Immune System

From the embryonic period, the development of the immune and nervous systems is regulated by each other. Immune cells are almost regulated by nerves from differentiation to function execution. And nerves perform hormone-mediated immune regulation through the hypothalamic-pituitary-adrenal (HPA) axis. The immune system also influences nerve growth and regulates the HPA axis through various immunoactive substances.

### 2.1 Neural Regulation of Immune Cell Production

Immune cells are originated from hematopoietic stem cells (HSCs) in the bone marrow, differentiate to the lymphoid stem cell stage which differentiate into pro-B and pro-T cells, and finally become a variety of immune cells in the bone marrow and thymus. However, studies have found that the nervous system regulates the generation, migration, and differentiation of HSCs directly or through substances such as neurotransmitters and neuropeptides (Agarwala and Tamplin, 2018).

The formation of HSCs occurs at the embryonic day 27–40, which refers to endothelial-to-hematopoietic transition (EHT) of hematopoietic endothelial cells of the embryonic dorsal aorta. Studies have found that hypoxic stress induces central neural crest cells to produce 5-hydroxytryptamine (5-HT) neurotransmitter signals in the embryonic zebrafish. 5-HT further affects the formation of distant embryonic hematopoietic stem and progenitor cells (HSPCs) *via* the HPA axis and local glucocorticoid (Kwan et al., 2016). Similarly, central cholinergic signals increase the secretion of catecholamines (CAs) and γ-aminobutyric acid (GABA) through the hypothalamic-pituitary-adrenal (HPA) axis and peripheral neurons, promoting the proliferation and mobilization of immature CD34^+^ HSCs (Spiegel et al., 2007; Pierce et al., 2017; Shao et al., 2021). In addition, peripheral neurons secrete CAs and calcitonin gene-related peptide (CGRP) to drive granulocyte colony-stimulating factor (G-CSF)-induced mobilization of HSCs (Katayama et al., 2006; Maryanovich et al., 2018; Gao et al., 2021).

### 2.2 Neural Regulation of Immune Cell Maturation and Release

The nervous system communicates with lymphatic tissues or organs through sensory and autonomic nerves. Among them, the thymus, as a central immune organ, receives dual innervation of sympathetic and parasympathetic nerves. Immunofluorescent staining of mouse thymus shows the inside nerve fibers distribution and indicates that T cell maturation is regulated by nerves (Al-Shalan et al., 2019). Lymph nodes are mainly supplied by sensory and sympathetic nerves. There are extensive and close contacts between nerve fibers and various dendritic cells in mouse lymph nodes (Hu et al., 2019). Sensory neurons detect the immune state of peripheral lymph nodes through nociceptors and undergo remodeling induced by inflammation, playing a vital role in inflammatory signal transduction and immune regulation (Huang et al., 2021). In the spleen, sensory nerves release neuropeptides to regulate the maturation, distribution, and function of immune cells (Felten et al., 1987; Jung et al., 2017), and denervation of the mouse spleen impairs plasma cell formation during T-cell-dependent immune responses (Zhang et al., 2020). The sympathetic nerve regulates the production of CXCL13 to organize the aggregation and distribution of immune cells in the spleen’s white pulp (Murray et al., 2017). Spleen cells of α7-nAChR knockout (α7-KO) mice produce more TNF-α and IL-6 (Fujii et al., 2017), suggesting that vagal nerve stimulation relieves systemic inflammation and prevents organ damage by inhibiting the production of cytokines in the spleen by acetylcholine (Ach) (Bassi et al., 2020).

### 2.3 Effect of the Immune System on Nerve

In bidirectional neuroimmune communication, immunogenic signals also act on neurons. As an important signal transduction factor of the immune system, cytokines not only coordinate the immune response but also mediate the signal exchange between the immune system and the nervous system, affecting the normal development of the nervous system and the activity of the neuroendocrine axis.

The early life inflammatory cytokine environment including maternal immune activation *in utero*, perinatal systemic inflammation, and childhood infection, represented by IL-1β, IL-6, TNF-α and IFN-γ, can affect the normal development of the nervous system (Jiang et al., 2018). This is probably due to the interaction of cytokines and major histocompatibility complex I (MHC-I) on neurons, and thus, negatively regulating synaptic plasticity and synaptic pruning (Paolicelli et al., 2011; Schafer et al., 2012). In addition, ciliary neurotrophic factor (CNTF), leukemia inhibitory factor (LIF), cardiotrophin-1 (CT-1), and IL-6 constitute a group of structurally related cytokines that is linked to neuronal nutrition and neuronal development through the NF-kb dependent pathway (Middleton et al., 2000).

Various cytokines have been shown to affect HPA axis activity *in vivo*. IL-1 increases adrenocorticotropic hormone (ACTH) and Glucocorticoid (GC) levels by influencing the secretory activity of the hypothalamus (Dunn, 2000). Moreover, various hematopoietic cytokines, such as TNF-α, IL-6, LIF, oncostatin M (OM), CNTF, interleukin-1 (IL-11), CT-1, etc., synergically enhance IL-1 mediated ACTH and corticosterone secretion to a certain extent (Turnbull and Rivier, 1999).

## 3 Neuroimmune Crosstalk in Skin, Lung, and Intestine

Skin, lung, and intestine share common anatomical features, including dense innervation and abundant resident immune cells. Nerve endings express cytokine receptors, and receptors for neuropeptides and neurotransmitters are also widely distributed on immune cells. Nerves and immune cells are close to each other in space. These points laid a physiological foundation for local neuro-immune interaction.

### 3.1 Neuroimmune Crosstalk in the Skin

The skin consists of epidermis, dermis, and subcutaneous tissue. Various inflammatory factors, neurotransmitters, neuropeptides, and receptors mediate neuroimmune communication between the epidermis, dermis, and appendages to regulate local skin homeostasis (Kobayashi et al., 2019) (Figure 1). The neuroimmune crosstalk of the skin plays a vital role in skin diseases such as psoriasis, atopic dermatitis, allergic dermatitis (Trier et al., 2019; Wang and Kim, 2020).

The nerves in the skin include the sensory, motor and autonomic nerves. Sensory nerves are divided into Aβ-fibers, Aδ-fibers, and C-fibers according to diameter and incoming velocity. C-fibers, including nociceptors and itch receptors, terminate in the epidermis and communicate most closely with neuroimmune (Lumpkin and Caterina, 2007). C-fibers are divided into peptide-energetic neurons and non-peptide-energetic neurons. The peptide-energetic neurons are marked by TRPV1 channels and can secrete neuropeptides, while the NP2 subpopulation of non-peptide-energetic neurons can also express TRPV1. The autonomic nerves of the skin are distributed only in the dermis, and most of them come from cholinergic sympathetic nerve fibers, which are involved in the blood and lymphatic circulation of the skin and the regulation of skin accessory organs. The autonomic nerves of the skin can secrete and release neurotransmitters such as acetylcholine, neuropeptides such as NPY, galanin, CGRP, and VIP, and neuromodulators such as tyrosine hydroxylase (Roosterman et al., 2006).

When sensing external noxious stimuli, skin sensory nerves regulate immunity by releasing neuropeptides, neurotransmitters, and other substances (Table 1) (Gouin et al., 2017). Immune cells also modulate sensory nerve activity by releasing inflammatory factors. Skin sensory nerves are divided into peptidergic neurons and non-peptidergic neurons. Peptidergic neurons marked by the transient receptor potential vanilloid1 (TRPV1) channel release a variety of neuropeptides, mainly Substance P (SP) and CGRP, occupying a leading position in skin neuroimmune (Roosterman et al., 2006). Mas-related G-protein-coupled receptor D-expressing (MrgprD) neurons in non-peptidergic neurons also inhibit mast cells (MCs) activation by releasing glutamate, thereby maintaining skin homeostasis (Zhang et al., 2021). The autonomic nerves of the skin are distributed only in the dermis, and they mainly consist of cholinergic sympathetic nerve fibers. The autonomic nerve participate in the blood and lymph circulation of the skin and the regulation of skin accessory organs by releasing neurotransmitters such as acetylcholine and neuropeptides (Roosterman et al., 2006).

**TABLE 1:** The role of neuropeptide in cutaneous neuroimmune crosstalk.

Substance	Main biological effect
SP	Activates MCs (INVALID CITATIONb; Subramanian et al. (2016); Asadi et al. (2012); Theoharides et al. (2010); Lim et al. (2017)
CGRP	Increases vascular permeability Krämer et al. (2005)
	Induces MCs degranulation Krämer et al. (2005)
	Promotes T helper 2 cells (Th2) responses suppressing T helper 1 cells (Th1) responses Krämer et al. (2005)
	Shifting langerhans cells (LCs) to type 2 responses Krämer et al. (2005)
NGF	Promotes nerve growth and secretion of SP and CGRP (Joachim et al. (2007); Roggenkamp et al. (2012); Blake et al. (2019a)
	Promotes immune cell activation and migration Skaper, (2017)
	Inhibits hair growth under stress (Peters et al., 2004)
VIP	Induces MCs degranulation Subramanian et al. (2016)
	Inhibits Th1 responses and nhances Th2 and T helper 17 cells (Th17) responses Ding et al. (2012)
	Increases vascular permeability Helyes et al. (2015)
NPY	Activates MCs Paus et al. (2006)
	Increases vascular permeability Paus et al. (2006)
Chemokine Like Family Member 4 (TAFA4)	Promotes macrophage production of IL-10, anti-inflammatory Hoeffel et al. (2021)
Norepinephrine	Controls innate immunity by stimulating T cells to secrete acetylcholine Rosas-Ballina et al. (2011)

Immune cells such as MCs, macrophages, neutrophils, basophils, T cells, and keratinocytes of the skin also act on neurons by releasing various active substances (Table 2).

**TABLE 2:** The reaction of immune cells to the nervous system.

Substances	Main biological effect
Histamine	Induces itch by TRPV1 neurons Peters et al. (2004)
5-HT	Activates HTR7: 5-hydroxytryptamine receptor 7 (HTR-7) to induce itch Morita et al. (2015)
IL-31	Causes itch by activating endothelin-1-responsive neurons to promote brain natriuretic peptide (BNP) synthesis and release Meng et al. (2018)
	Activates TRPV1+ neurons (Cevikbas et al., 2014; Xu et al., 2020a)
	Promotes GRP release to induce itch Sakata et al. (2019)
IL-1	Promotes SP release Baral et al. (2019)
	Causes pain receptor sensitization Baral et al. (2019)
IL-33	Causes itch Franke et al. (2021)
	Increases the release of vascular endothelial growth factor (VEGF) and TNF from MCs with SP Theoharides et al. (2010); Taracanova et al. (2017)
IL-4, IL-13	Induces chronic itch Oetjen et al. (2017)
IL-6	Causes pain receptor sensitization (Baral et al. (2019)
IL-10	Inhibits pain neuron activation Baral et al. (2019)
IL-17	Induces TRPV4 expression to mediate mechanical hyperalgesia Segond von Banchet et al. (2013)
	Causes pain receptor sensitization Baral et al. (2019)
TNF	Causes pain receptor sensitization Baral et al. (2019)

#### 3.1.1 Immune Crosstalk of Pruritus Receptors

Nerve endings that transmit itch and pain communicate most closely with immune cells and are highly associated with inflammation (Wang and Kim, 2020).

Most pruritus receptors are non-peptidergic (NP) neurons, consisting of three subgroups: NP1, NP2, and NP3. Each subgroup expresses different receptors and channels. Itch-inducing cytokines are often associated with type 2 cell responses. Keratinocytes release cytokines such as transient receptor potential ankyrin 1 (TSLP), which activate downstream type 2 immunity (Tamari et al., 2021). Type 2 immune cells release histamine, cytokines (IL-4, IL-31, IL-13, IL-33, 5-HT, TSLP), 5-nitro-2-(3-phenylpropylamino)-benzoic acid (NPPB), and other itch factors to activate TRPV1 and transient receptor potential ankyrin 1 (TRPA1), which in turn activate Voltage-gated Na (+) (NaV) channels. Thus, action potentials are generated, and the signals are sent to the brain to generate itch. Itch causes scratching behavior, which make keratinocytes damaged to release TSLP, activating downstream pathways and forming a vicious circle (Wilson et al., 2013a; Mack and Kim, 2018).

Itch is closely related to MCs. In pathological conditions such as psoriasis and epidermal hyperplasia, MCs are increased and located near the primary afferent nerve endings that transmit itch and pain sensations, and there is bidirectional solid crosstalk between them (Voss et al., 2021). On the one hand, MCs release various mediators to activate pruritus receptors through direct or indirect pathways; on the other hand, MCs also generate nerve growth factor (NGF) to promote the growth of sensory nerve fibers (Leon et al., 1994). Furthermore, innervation is also necessary for MCs function. Passive cutaneous anaphylaxis (PCA)-induced degranulation of MCs is significantly reduced under denervation of sensory neurons (Siebenhaar et al., 2008).

Other immune cells are also involved in the generating of itch. Neutrophils activate C-X-C motif chemokine receptor 3 (CXCR3) by upregulating the expression of C-X-C Motif Chemokine Ligand 10 (CXCL10), leading to sensory nerve activation (Walsh et al., 2019). In wounds, dendritic cells, especially type 2 conventional dendritic cells (cDC2s), are the primary source of IL-31. IL-31 induces the expression of TRPV1 and Interleukin 31 Receptor A (IL-31RA) in neurons through the Jak1-Stat3 pathway, thereby increasing the sensitivity of itch sensory neurons (Xu et al., 2020a). Eosinophils, which rise rapidly after skin exposure to toxicants, mediate inflammation and are also able to alter local innervation and promote itch by increasing SP expression (Lee et al., 2015).

#### 3.1.2 Immune Crosstalk of Painful Nerve Endings

Immune cells release cytokines (such as TNF-α, IL-6, IL-17A, IL-1β, NGF, prostaglandin E2), lipids, and other factors. These factors directly activate neurons and lead to the production of pain. And they also indirectly cause pain sensation by causing sensitization of central or peripheral neurons (Cook et al., 2018). Cytokines involved in pain are often associated with type 1 or 17 cell responses.

Macrophages are closely connected with pain perception. After tissue injury or infection, macrophages release various chemokines and lipid mediators. These factors bind to receptors on nociceptors, leading to increased activity of TRPA1/TRPV1 and voltage-gated sodium channels of nociceptors through activation of the downstream mitogen-activated protein kinases (MAPK) pathway and protein kinase A (PKA), resulting in increased pain sensitivity. The activation of nociceptors lead to the release of SP, which binds to macrophage neurokinin-1 receptor (NK-1R), leading to the infiltration of macrophage cells, forming positive feedback (Chen et al., 2020a). In addition, monocytes and macrophages also play an analgesic effect by releasing anti-inflammatory factors such as IL-10 and specialized pro-resolving mediators (SPMs) (Ji et al., 2016). Macrophages also express neurotrophic factors such as NGF and brain derived neurotrophic factor (BDNF), which maintain neuronal growth and survival (Blake et al., 2019a).

Keratinocytes are also closely related to pain receptors. Under physiological conditions, keratinocytes play analgesic and rewarding roles by being able to release β-endorphin, a proopiomelanocortin (POMC)-derived peptide (Fell et al., 2014). However, when keratinocytes are damaged, it leads to hyperalgesia. Pain neurons also release neuropeptides, leading to further activation of keratinocytes, which promotes the development of inflammation (Talagas et al., 2020).

Pain neurons modulate immune cell activity by releasing mediators such as neuropeptides and even miRNA-rich exosomes (McMahon et al., 2015; Simeoli et al., 2017; Baral et al., 2019; Huang et al., 2021). Activation of nociceptors also initiates or enhances neurogenic inflammation (Michoud et al., 2021). During infection, pathogens activate pain receptors through pattern recognition receptors (PRRs), resulting in the release of neuropeptides and the activation of immune cells. And they also directly activate the immune system to activate pain neurons through inflammatory factors. The nervous system and the immune system activate each other and work together to strengthen the defenses (Chiu, 2018).

Pain also leads to neuron-mediated immunosuppression. Nav1.8 is a sodium channel, mainly expressed in C-fibers of sensory neurons in the dorsal root ganglia. During HSV-1 infection, Nav1.8 + nociceptors inhibit skin neutrophil infiltration, limit tissue damage, control dendritic cells (DCs) responses to antigen presentation, and induce the initiation of CD8^+^ T cells (Filtjens et al., 2021).

#### 3.1.3 Neuroimmune Crosstalk in Cutaneous Host Defense

Skin nerves play an integral role in regulating host defenses. Pathogen molecules such as endotoxin and flagellin directly activate receptors on sensory neurons to induce pain or itch to modulate immune responses (Chiu et al., 2013a; Chiu et al., 2013b). In response to nociceptive stimuli, inflammatory factors, and pathogen molecules, TRPV1+ neurons specifically activate the type 17 immune response by releasing CGRP to stimulate DCs to produce IL-23. IL-23 prompts γδT cells to produce IL-17 and IL-22 cytokines, which in turn recruit neutrophils and monocytes to enhance host defense (Cohen et al., 2019). This means that nerve fibers in the inflamed skin can activate the immune defenses of the uninfected skin, enabling it to fight against the potential threat of infection (Kashem et al., 2015a; Cohen et al., 2019). This pathway enhances the skin’s defense against C. Albicans (Kashem et al., 2015b) and leads to psoriatic dermatitis (Riol-Blanco et al., 2014). During HSV-1 infection, Nav1.8 + nociceptors are activated to inhibit skin neutrophil infiltration, limit tissue damage, and control DC responses to affect antigen presentation and induce the initiation of CD8^+^ T cells (Filtjens et al., 2021).

Nevertheless, it is worth noting that neuroimmune does not just boost defenses. Pathogens also promote self-transmission by suppressing pain perception through neuroimmune pathways or activating pain to suppress the immune system (Baral et al., 2019). In necrotizing fasciitis, *Streptococcus* pyogenes secrete streptolysin S (SLS) to activate neurons to generate pain and cause pain neurons to release CGRP. CGRP inhibits the recruitment of neutrophils (Chiu et al., 2013a) and the release of TNF-α from macrophages, which reduces immune bactericidal effect (Chiu, 2018).

#### 3.1.4 Neuroimmune Crosstalk in Skin Inflammation

Skin neurogenic inflammation is caused by the neuropeptides released from sensory nerve endings, manifested as vasodilation, plasma extravasation, edema, cell infiltration, and enhanced nociception (Peters et al., 2005; Sorkin et al., 2018). Neurogenic inflammation promotes skin healing and immune defense but may increase pathological immune responses such as anaphylaxis.

The neuroimmune interaction between peripheral nerve endings and MCs is critical in neurogenic inflammation. After peripheral nerve stimulation, various factors are produced to drive MCs degranulation, which in turn recruits immune cells such as monocytes, eosinophils, and neutrophils (Kulka et al., 2008). SP activates immune cells, causing the release of leukotrienes through the lipoxygenase pathway. Leukotrienes cause plasma extravasation, leading to neurogenic edema (Le Filliatre et al., 2001). SP and CGRP can also act on vascular endothelial cells and smooth muscle cells, increasing vascular permeability and increasing plasma extravasation (Saria, 1984; Brain and Williams, 1989).

Neuropeptides such as SP and CGRP released from nerve endings activate MCs to release mediators such as histamine, leading to the aggregation of inflammatory cells. In turn, MCs and immune cell product activate neurons (Siebenhaar et al., 2008; Steinhoff et al., 2018). Neural and immune promote each other, which forms a vicious circle leading to the generation of neurogenic inflammation (Rosa and Fantozzi, 2013). Interactions between neuropeptides, neuropeptide receptors, and neuropeptide-degrading enzymes are key for the progression of neurogenic inflammation (Cattaruzza et al., 2009).

#### 3.1.5 Neuroimmune Crosstalk in Skin Allergic Diseases

In allergic skin reactions, allergens directly activate TRPV1+ sensory neurons, and results in itch and pain (Perner et al., 2020). TRPV1+ nerve fibers colocalize closely with MRGPRB+ (MAS Related GPR Family Member D) MCs in the skin. During allergy, activated TRPV1+ nerve fibers activate MRGPRB2 on MCs through SP, leading to the release of inflammatory factors (Serhan et al., 2019a; Serhan et al., 2019b). SP also activate MRGPRA1(Mas-related G-protein coupled receptor member A1) on CD301b + DCs, causing their migration to dLN to initiate Th2 cell differentiation and induce skin type 2 inflammation (Perner et al., 2020). The recruited inflammatory cells produce a large number of cytokines, which further activate neurons to generate itch and pain with releasing neuropeptides. Allergens can also directly activate basophils to produce leukotrienes, especially Leukotriene C4 (LTC4) (Chen et al., 2020b). Leukotrienes lead to acute pruritus in atopic dermatitis (AD) through CysLTR2 (Cysteinyl Leukotriene Receptor 2) receptors on a subset of NP3 sensory neurons (Wang et al., 2021). Itch induces scratching behavior, which leads to the release of TSLP from damaged skin keratinocytes. TSLP leads to the aggravation of type 2 inflammation, thus forming a vicious circle (Wilson et al., 2013b; Buhl et al., 2020). Keratinocytes are also capable of secreting NGF and glial cell-line-derived neurotrophic factor (GDNF) to induce neurite outgrowth and increase CGRP + sensory fibers, increasing nerve fiber density in the epidermis of patients with atopic eczema (Roggenkamp et al., 2012).

### 3.2 Neuroimmune Crosstalk in the Lungs

In the lungs, sensory nerves are distributed in every layer of the airway, especially in the mucosal layer of the airway. Their nerve endings form some special structures, such as nociceptors and neuroepithelial cell body (NEB) (Gelb and Nadel, 2016; Mazzone and Undem, 2016). Sympathetic and parasympathetic nerves enter the lung with blood vessels and bronchus and are distributed around smooth muscle, glands and vessels (Figure 2). Dense nerve distribution facilitates the regulation of airway tension, mucus secretion, and cough reflex, and resident immune cells make a timely immune response to external stimuli. Epithelial cells, neurons, and immune cells interact to maintain the relative homeostasis of the lungs.

Sensory innervation of the respiratory tract mainly comes from the vagus branch from nodose/jugular ganglia and the somatosensory afferent nerves comes from neurons in the dorsal root ganglion (Camp et al., 2021). Pulmonary sensory nerve endings express Toll-like receptors (TLRs, a type of pattern-dependent recognition receptor) that recognize pathogen-associated molecular patterns (PAMPs), such as lipopolysaccharides (LPS, a component of the cell wall of gram-negative bacteria), and transmit signals to the central nervous system (CNS). The CNS makes a comprehensive response and sends signals through the vagus nerve to increase the expression of acetylcholine (Ach), promote cough reflex and regulate immune defense (Chen et al., 2020b; Yamada and Ichinose, 2018a; Jung et al., 2018). Also, pulmonary sensory nerve fibers constitute special nociceptors, which, under the stimulation of allergens and pathogens, on the one hand, cause cough, pain, and bronchoconstrictive reflex for initial resistance, on the other hand, induce neurogenic inflammation through calcium-mediated neuropeptide release (Table 3) (Chen et al., 2020b; Baral et al., 2018). This process is clinically common in allergic diseases, asthma, chronic obstructive pulmonary diseases (COPD), etc., Meanwhile, cytokine receptors such as IL-5R are also expressed in the sensory nerve endings, receiving cytokine signals in the inflammatory response, producing positive feedback and enhancing immunity (Huh and Veiga-Fernandes, 2020). In addition, another sensor, the NEB, is also distributed in the airway. They are compact structures within the mucous membrane of the airway and are innervated by vagal afferent fibers. The apical surface of NEB is exposed to the airway, responding to a series of stimuli such as hypoxia, hypercapnia, mechanical stretching, norepinephrine, etc., and producing several bioactive mediators, including bell bombesin, CGRP, and 5-HT (Table 3) (De Virgiliis and Di Giovanni, 2020; Mahmoud et al., 2021). Sensory nerves from the dorsal root ganglion can not only release neuromedin U (NMU) to promote the proliferation of ILC2 and type 2 inflammatory response (Cardoso et al., 2017; Klose et al., 2017; Wallrapp et al., 2017), but also secrete neuromedin B (NMB) to inhibit ILC2 response, eosinophilia, and mucus production in the stage of allergic inflammation and worm infection, forming a bidirectional regulation (Chen et al., 2020b; Inclan-Rico et al., 2020).

**TABLE 3:** The role of neuropeptide in pulmonary neuroimmune crosstalk.

Substance	Main biological effect
SP	Promotes bronchoconstriction Lo et al. (2018)
	Increases mucus secretion Talbot et al. (2020)
	Increases the release of cytokines from MCs (Thapaliya et al. (2021); Wang et al. (2022)
	Promotes immune migration, increases neutrophil adhesion and phagocytic activity, and stimulates the virulence of *Staphylococcus aureus* Mashaghi et al. (2016)
CGRP	Promotes bronchoconstriction and vasodilation Lo et al. (2018)
	Stimulates ILC2s and downstream immune responses Sui et al. (2018)
VIP	Relaxes airway smooth muscle and pulmonary resistance vessels Szema et al. (2017)
	Increases gland secretion McMahon et al. (2020)
NMB	Inhibits the type II inflammatory response (Chen et al., 2020b; Inclan-Rico et al., 2020)
NMU	Activates ILC2s and amplifies il-25-induced allergic inflammation (Wallrapp et al. (2017); Xu et al. (2020b)
	Promotes Th2 cytokine production and type 2 inflammatory tissue response Cardoso et al. (2017)

Cholinergic parasympathetic nerves originate from the vagus nucleus of the medulla oblongata and function through the release of ACh (Huang et al., 2019). Binding to M1/M3 receptors, Ach causes a pro-inflammatory effect, promoting bronchoconstriction, mucus secretion and vasodilation, and participating in the cough reflex, which is closely related to asthma, and atopic diseases (Cazzola et al., 2021). However, the effect of Ach on the N receptor is mostly inflammation-resisting. Immune cells express α7 nicotinic acetylcholine receptor (α7nAChR), which binds to Ach to suppress the release of inflammatory cytokines. This specific pathway is known as the cholinergic anti-inflammatory pathway (Yamada and Ichinose, 2018a). For smokers, nicotine, the main component of cigarettes, is the ligand of nicotinic acetylcholine receptor (nACHR), therefore possesses anti-inflammatory properties (Bosmans et al., 2017).

Sympathetic innervation of the lungs originates from the sympathetic ganglion generated from the upper thoracic segment of the spinal cord, and acts on bronchial vessels and submucosal glands through norepinephrine (NE), mainly regulating bronchodilation and mucus secretion under pressure (De Virgiliis and Di Giovanni, 2020). NE inhibits the release of cytokines through β2 adrenergic receptors (β2AR) of immune cells and plays an anti-inflammatory role.

In addition, there are several special cells in the lungs, which are significant in neuroimmune, leading to many pathological states. Pulmonary neuroendocrine cells (PNECs) are endodermal-derived airway epithelial cells innervated by sensory nerves, which are enriched in airway branch points and collect external environmental information from the air (Xu et al., 2020b). PNECs release neuropeptides and neurotransmitters under hypoxia, hypercapnia and nicotine stimulation to regulate inflammatory response, and mucus secretion of airways (Garg et al., 2019). Neuroairway associated macrophage (NAM) is a newly discovered special mesenchymal tissue-resident macrophage, which is closely related to neuronal projection in the airway. It is found that NAM reduces inflammatory damage in influenza virus infection, but the specific mechanism remains unclarified (Ural et al., 2020). Whether NAM participates in the resistance of Severe Acute Respiratory Syndrome Coronavirus 2 (SARS-CoV-2) infection and its possible clinical application is currently a research hotspot (De Virgiliis and Di Giovanni, 2020). Eosinophils and lymphocytes in the lungs synthesize neurotrophic factors (NGF and BDNF) that bind to receptors (TrkA/B) to increase nerve length and branch points, exposing nerve endings to lumen for storage and release of neurotransmitters and neuropeptides from these TRPV1 + fibers. It increases mucus secretion, collagen deposition, and smooth muscle hyperplasia (Pavón-Romero et al., 2021).

#### 3.2.1 Neuroimmune Crosstalk in the Pulmonary Immune Defense Response

It has been found that sensory nerves play a major role in pulmonary infectious diseases (releasing neuropeptides to initiate immune defense (Table 3)). In the case of *Staphylococcus aureus*, SP stimulates bacterial virulence and promotes the migration, adhesion, and phagocytic activity of neutrophils. TRPV1+ afferent nerve in nociceptor modulates protective immunity by releasing cGRP, inhibiting neutrophil recruitment and γδT cell-mediated defense (Baral et al., 2018; Camp et al., 2021). In helminth infection, NEB can release NMU and NMB to bidirectionally regulate the immune response. NMU activates the second group of innate lymphocytes (ILC2)-mediated type 2 inflammatory response and aggravates inflammatory damage (Cardoso et al., 2017; Xu et al., 2020b). NMB inhibits ILC2 proliferation and reduces inflammation (Inclan-Rico et al., 2020).

Many studies on SARS-CoV-2 suggested that its approach to human host cells is mainly mediated by transmembrane protein angiotensin-converting enzyme 2 (ACE2) and transmembrane protease serine 2 (TMPRSS2), and these receptors are highly expressed in airway epithelium and pulmonary parenchyma (De Virgiliis and Di Giovanni, 2020). Patients are often accompanied by cerebral injury symptoms, such as headache, nausea and vomiting, confusion of consciousness, epilepsy, etc., (Acharya et al., 2020). Studies have revealed that SARS-CoV-2 spreads to the choroid plexus through blood transmission and the breakthrough blood-brain barrier to infect the CNS (Matsuda et al., 2004; Desforges et al., 2019; Fagre et al., 2021). In Corona Virus Disease 2019 (COVID-19), upregulated inflammatory mediators interact with receptors expressed on sensory neurons to activate sensory neurons and release neuropeptides, leading to vasodilation, immune cell recruitment, and neurogenic inflammation (Song et al., 2021). In addition, the newly discovered NAM is proved to relieve inflammation in influenza and has the possibility of fighting the inflammatory storm in severe cases of COVID-19 (Schneider et al., 2014).

#### 3.2.2 Neuroimmune Crosstalk in Pulmonary Inflammation

COPD is a classic type of pulmonary airway inflammation characterized by persistent respiratory symptoms and airflow restriction. We take COPD as an example to illustrate the neuroimmune crosstalk common in pulmonary inflammation.

COPD is developed from chronic bronchitis and emphysema to the stage of continuous airflow limitation in pulmonary function examination, and its incidence and prevalence remain high in China. Increased activity of the cholinergic system in COPD results in airway smooth muscle contraction leading to airflow restriction (Yamada and Ichinose, 2018b). The development of COPD goes through a long inflammatory progression stage, during which the time for Ach release is prolonged, the expression of M1R and M3R in airway structure is increased, and the pro-inflammatory effect of Ach through the M receptor is enhanced (Blake et al., 2019b). While α7nAChR inhibits the release of inflammatory cytokines by inflammatory cells (including macrophages and dendritic cells) and has a pro-inflammatory effect during the inflammatory stage (Hollenhorst and Krasteva-Christ, 2021). During acute inflammation, neuropeptides released by sensory nerve endings modulate the progression of inflammation. SP increases adherent aggregation and phagocytic activity of neutrophils. VIP reduces the contraction response of airway smooth muscle and reduces the degree of airflow restriction (Table 3) (Camp et al., 2021).

#### 3.2.3 Neuroimmune Crosstalk in Asthma

Asthma is a recurrent disease characterized by chronic airway inflammation and airway hyperresponsiveness. Allergens enter the airway and activate T cells through antigen presentation. Activated helper Th2 cells secrete cytokines such as interleukin to directly activate MCs and eosinophils, leading to chronic airway inflammation. B cells can also be activated to produce specific IgE, which binds to MCs and eosinophils to cause smooth muscle contraction. Several neuroimmune processes are involved in the development of asthma.

IL2C is important in allergic diseases. It is regulated by a variety of neuropeptides and neurotransmitters and produces massive cytokines to regulate the immune response. Nociceptors, NEB, and PNECs innervated by sensory nerves receive allergen stimulation and immediately induce the secretion of neuropeptides (VIP, CGRP, SP, etc.,) to cause neurogenic inflammation (Table 3) (Mou et al., 2021). VIP and CGRP enhance the activity of ILC2, TH2, and other cells (Nassenstein et al., 2018), and promote the production of inflammatory factors. SP promotes the degranulation of MCs, release of pro-inflammatory cytokines, and immune migration, increasing the adhesion of neutrophils to bronchial epithelial cells and neutrophils phagocytic activity. In addition, CGRP directly activates ILC2s at a close spatial distance under allergen stimulation, not only triggering downstream type 2 immune response but also promoting ILC2s to produce IL-5, recruiting eosinophils, and aggravating allergic reactions (Camp et al., 2021). PNECs amplify inflammation by releasing CGRP and the neurotransmitter GABA. Among them, GABA induces the hyperplasia of airway epithelial goblet cells and causes excessive secretion of airway mucus (Sui et al., 2018).

The parasympathetic nerve mainly mediates smooth muscle contraction and cough reflex through Ach, exacerbating dyspnea in asthmatic patients (Gosens and Gross, 2018). Ach binds to muscarinic acetylcholine receptor (mAChR) on dendritic cells to promote the polarization of TH2 cells and binds to MCs to increase their activity and aggravate airway edema and exudation (Bosmans et al., 2017). Ach acts on N receptors of immune cells to reduce the production of TSLP, thereby inhibiting the activity of ILC2s, promoting the immunosuppression of Treg cells, reducing the degranulation and cytokines of MCs, and cutting down the release of histamine and migration of eosinophils from basophils. In addition, a7nAChR inhibits the production and release of inflammatory factors on immune cells to cause a cholinergic anti-inflammatory effect, which relieves asthma airway to a certain extent. For example, Ach combined with a7nAChR on ILC2s reduces the proliferation of ILC2, suppresses the production of IL-5 and IL-13, and aggravates ILC2-mediated airway hyperresponsiveness (AHR) (Yamada and Ichinose, 2018a). Macrophage a7nAChR inhibits the inflammatory mediators, such as TNF-α and IL-1β, to produce and reduce inflammatory damage by binding Ach. Anticholinergic and muscarinic receptor antagonists are applied to relieve bronchoconstriction in asthma (Bosmans et al., 2017).

Under stress, NE produced by sympathetic nerve activation binds to β2AR can not only relax smooth muscle but also inhibit ILC2 mediated type 2 inflammation and mucus production, alleviating asthma symptoms. Studies have found that β2AR agonists effectively relax smooth muscle relaxation, inhibit immune cell recruitment, and cytokine release (Voisin et al., 2017).

### 3.3 Neuroimmune Crosstalk in the Intestinal Tract

Neurons in the intestinal tract are divided into the extraintestinal nerves and the enteric nervous system (ENS) by their location (Furness, 2012). Extraintestinal innervation includes the sympathetic nerve, vagus nerve, dorsal root nerve and nociceptive nerve. Neurons of ENS are mainly located in the submucosa and muscular layer, and together with glial cells form a ganglion network surrounding the intestinal tube, which is spatially divided into two layers: the intramuscular Austenian plexus and the submucosal plexus (SMP) (Furness, 2008; Schemann et al., 2020). ENS forms reflex circuits with the external sympathetic and parasympathetic nervous systems that innervate all intestinal layers, mediating intestinal motility, epithelial secretion, and vascular regulation (Benarroch, 2007; Schneider et al., 2019). Similarly, the intestinal tract is also rich in resident immune cells, which are mostly clustered in the lamina propria and muscularis of the mucosal area, including innate immune cells (macrophages, MCs, NK cells, innate lymphocytes, γδT cells, etc.) and adaptive immune cells (T cells, B cells). This anatomical intimacy between neurons and immune cells provides the basis for neuro-immune interaction (Figure 3).

Gut sensory nerves receive nutrients, mechanical stretching, lumen pressure, and immune stimulation to carry gut sensory signals from the gut to the brain stem and spinal cord (Spencer and Hu, 2020). They recognize harmful and inflammatory stimuli in three ways:

1) PRRs, such as TLRs, to recognize PAMPs produced by invading bacteria or viruses during infection (Barajon et al., 2009; Burgueño et al., 2016);
2) Cytokine receptors, to recognize factors secreted by immune cells (IL-1β、IL-6、IL-10 and TNF-α, etc.,) (Hughes et al., 2013);
3) Danger signal receptors, especially fast electrical channels (TRPV1, TRPV4, TRPA1, and TRPM8), to recognize oxidative stress products (ATP, uric acid, hydroxynonenal, etc.,) during harmful stimuli (heat, acid, and chemicals, etc.,), inflammation and tissue damage (Reinshagen et al., 1994).

Activated sensory nerves, on the one hand, produce reverse axonal reflex. It promotes the release of neuropeptides (SP, CGRP, VIP, etc.,) from nerve terminals on resident immune cells, leading to chemotaxis and activation of neutrophils, macrophages, MCs and lymphocytes (Table 4). At the same time, these neuropeptides can directly act on vascular endothelial cells, thereby promoting vascular dilation and increasing capillary permeability, resulting in plasma extravasation and edema. On the other hand, the activated nervous system produces cytokines to participate in immune activities (Kermarrec et al., 2019; Jarret et al., 2020).

**TABLE 4:** Regulation of intestinal neuropeptides on immunity.

Neuropeptide	Role in gut
SP	Activates the NF-KB pathway of target cells (macrophages, MCs, etc.)
	Promote the release of pro-inflammatory factors IL-1 β, IL-6, IL-8, TNF-α Shimizu et al. (2008)
CGRP	Antagonizes the proinflammatory effect of SP Engel et al. (2012b)
	Promotes prostacyclin (PGI2) synthesis, reduces TNF-α production, and inhibits neutrophil aggregation Okajima and Harada, (2006)
VIP	Reduces the number of cells expressing TLR2 and TLR4, and decreases CD4+T cells in lesions Arranz et al. (2006)
	Inhibits the production of CXCL10 and promotes the production of C-C motif chemokine 22 (CCL22), thereby shifting the immune response to Th2 and reducing Th1 cell infiltration in inflammatory lesions Delgado et al. (2004a)
	Inhibits the production of pro-inflammatory cytokines and promotes the production of IL-10 and IL-1 receptor antagonists (IL-1Rα), thereby mediating anti-inflammatory effects Delgado et al. (2004b)
	Activates the secretion of interleukin-22 (IL-22) in group 3 innate lymphoid cell (ILC3s) to promote the mucosal barrier function Seillet et al. (2020)
PACAP	Anti-inflammatory effect
	Promotes the balance of Th1/Th2 reaction Azuma et al. (2008)

The immune system also acts as a counterforce to the intestinal nervous system. Cytokines are the main signaling molecules, which regulate the intestinal nerve density and function. For example, TNF-α and IL-1β promote neurite growth by increasing glial secretion of GDNF (INVALID CITATIONa), while IL-1β and IL-6 modulate synaptic sensitivity and transmission efficiency (Xia et al., 1999).

#### 3.3.1 Neuroimmune Crosstalk in Intestinal Host Defense

As mentioned above, sensory nerves densely distributed in the gut sense PAMPs and products and actively participate in host defense by releasing neuropeptides (SP, CGRP, VIP, etc.,) (Lai et al., 2017; Mao et al., 2013). On the one hand, these neuropeptides interact with immune cells to mediate neurogenic inflammation, intestinal contraction and mucus secretion, and is important for pathogen clearance (Table 5). On the other hand, due to the similar structure to antibacterial peptides (AMP), neuropeptides act on the negatively charged cell membrane of bacteria, change the membrane structure through depolarization, produce physical pores, and play an antibacterial role (Ganz, 2003; Hansen et al., 2006).

**TABLE 5:** The role of neuropeptides in intestinal host defense.

Neuropeptide	Role	Pathogen
SP	Activates macrophages, promotes IL-12 production, and further induces IFN-γ to mediate pathogen clearance (Kincy-Cain and Bost. (1996); Kincy-Cain and Bost. (1997)	*Salmonella*
	Promotes secretory immunoglobulin (S-IgA) response Walters et al. (2005)	
CGRP	Reduces the density of intestinal micro pleated cells (M cells) and maintains the level of small intestinal filamentous bacteria (SFB), mediating *salmonella* infection Lai et al. (2020)	*Salmonella*
VIP	Promotes ILC3 recruitment and enhances IL-22 signaling, mediating protection against bacterial infections Yu et al. (2021)	C.rodentium

In addition, the intestinal nervous system can produce cytokines directly involved in host defense. Intestinal intermuscular plexus neurons produce IL-18 and stimulate goblet cells to secrete AMP and mucus, thereby enhancing the protective effect of intestinal mucus barrier and mediating protection against bacterial infection (*Salmonella*) (Jarret et al., 2020).

However, neuro-immune bi-directional communication during host defense is a double-edged sword. Persistent low-grade inflammation of the intestinal tract after infection leads to sensitization and loss of intestinal neurons, which is an important pathogenic factor of intestinal dysfunction after infection. There is an increased risk of irritable bowel syndrome after intestinal infection (Halvorson et al., 2006; Spiller and Garsed, 2009). Many patients have long-term abdominal pain after infection, which may be related to chronical sensitization of TRPV1+ neurons in the intestinal tract (Balemans et al., 2017). In addition, the non-classical inflammasome NlrP6 and caspase-11 (Casp11) -dependent pathway mediates rapid and sustained loss of intestinal neurons in the persistent hypoinflammatory state of postinfection, which is associated with intestinal motility disorders (Matheis et al., 2020).

In contrast, neuroimmunity in the gut also mediates anti-inflammatory responses and tissue damage repair during homeostasis. Tyrosine hydroxylase+ (TH+) neurons signal to myoclastic macrophages (MM) *via* β2AR to polarize MM into a tissue-protective phenotype (Gabanyi et al., 2016). During the intestinal pathogen infection, these macrophages protect intestinal neurons from Casp11-dependent death by expressing arginase and protective polyamines, which are important tissue-protective effects during postinfection and homeostasis (Matheis et al., 2020; Ahrends et al., 2021).

#### 3.3.2 Neuroimmune Crosstalk in Inflammatory Bowel Disease

During intestinal inflammation, activated neurons release neurotransmitters and neuropeptides that play a pro-inflammatory or anti-inflammatory role and regulate inflammatory and immune states.

The effect of adrenergic neurons on inflammation depends on receptor binding ability. At low concentrations, NE binds *a* -adrenergic receptors, while at higher concentrations, NE primarily interacts with β receptors (Straub et al., 2006; Willemze et al., 2019). *a* receptors have pro-inflammatory effects and can promote the production of downstream pro-inflammatory cytokines, which play a significant role in intestinal defense function (Green et al., 2003). Blocking α2-adrenergic receptors down-regulate TNF and IL-1β and improve 2,4,6-trinitrobenzenesulfonic acid (TNB)-induced colitis (Bai et al., 2009). In contrast, several experiments have shown that exogenous NE can act on β receptors and exert dose-dependent anti-inflammatory effects.

They can inhibit the expression of macrophage inflammatory cytokines IL-1β, IL-6, IL-12, TNF-α in the inflammatory state, thereby alleviating colon inflammation and edema symptoms (Willemze et al., 2019; Mallesh et al., 2021). In the inflammatory state of the intestinal mucosa, semaphorin 3C (SEMA3C) and other sympathetic rejection factors are highly expressed, the intestinal sympathetic nerve fibers are lost (Straub et al., 2008), and the synthesis and storage of NE, DA and 5-HT are blocked (Magro et al., 2002). Besides, the density of *a* receptors is increased and of β receptors is decreased (Martinolle et al., 1993; Blandizzi et al., 2003). All this evidence suggests that imbalance of sympathetic energy transmission may be a part of IBD.

The vagus nerve has an important protective role in IBD, including acute recurrence of experimental colitis and chronic colitis (Ghia et al., 2006; Ghia et al., 2007; Liu et al., 2020). A nationwide cohort study in Sweden showed that patients undergoing vagotomy had an increased risk of developing IBD and that vagotomy was positively associated with later IBD (Liu et al., 2020). In the intestinal tract, the vagus nerve inhibits monocyte infiltration and macrophage activation, and reduces the release of related inflammatory factors (IL-1β, TNF-α, INF-γ, IL-18, etc.,), thereby inhibiting early acute inflammatory response and improving mucosal epithelial integrity (Ghia et al., 2006; Ghia et al., 2007). In addition, sacral nerve stimulation has been shown to improve colonic inflammation and enhance colonic barrier function by enhancing parasympathetic nerve activity and modulating ENS and immune system activity in mice (Tu et al., 2020). Conversely, vagal inhibition and decreased acetylcholine levels increase susceptibility to intestinal inflammation (Ghia et al., 2008). The activity of NF- kB in colonic mucosa and the expression of inflammatory factors such as IL-1β and TNF-α in colonic macrophages of mice with vagal nerve resection were significantly increased (Ghia et al., 2006; O'Mahony et al., 2009).

Sensory neuro-immune communication is also important in the development and progression of IBD (Kihara et al., 2003; Kimball et al., 2004; Engel et al., 2011; Engel et al., 2012a). Multiple pieces of evidence have confirmed the pro-inflammatory role of TRPV1+ and TRPA1+ neurons in experimental colitis. TLR4 co-localization with TRPV1 has been observed in a variety of primary afferent neurons, including DRG. In the course of colitis, the expression of TLR4 in DRG is increased, which upregulates TRPV1 expression and TRPV1 current density, mediates inflammatory pain, and regulates inflammatory state (Shen et al., 2017; Wu et al., 2019; Esquerre et al., 2020). Furthermore, the severity of colitis was positively correlated with the gradient of TRPV1 positive neurons in the colon (Engel et al., 2012a). This may be due to the activation of sensory neurons by inflammatory mediators that sensitize TRPV1 and TRPA1 channels, promote Ca2+ channel opening, and release pro-inflammatory neuropeptide SP, leading to pro-inflammatory events (Engel et al., 2011; Engel et al., 2012a; Bertin et al., 2014). TRPM8+ neurons play a protective role in acute colitis (innate immune cell-mediated) (Ramachandran et al., 2013; de Jong et al., 2015). TRPM8 is located in mucosal epithelial cells and high-threshold colonic sensory neurons (Austroy’s plexus) (Ramachandran et al., 2013), which may co-express TRPV1 and have the ability to cross-desensitize TRPV1 (Brain and Williams, 1989). Activation of TRPM8 blocks TRPV1-mediated calcium signaling, thereby inhibiting inflammation (Ramachandran et al., 2013).

#### 3.3.3 Neuroimmune Crosstalk in Food Allergy

Food allergy is mainly manifested as hypersensitivity reaction (type I) caused by the interaction between submucosal sensory neurons and MCs. It is characterized by increased mucosal secretion and intense contraction of muscle tissue, leading to abdominal pain and diarrhea symptoms (Schemann and Camilleri, 2013; Yashiro et al., 2021).

In allergic reactions, specific IgE binding to FCεR1 on the MC surface recognizes the allergen, degranulates after activation, and releases mixed mediators (histamine, prostaglandin, leukotriene, 5-HT, cytokines, complement, etc.,) (Liu et al., 2003; Van Nassauw et al., 2007). Histamine is an important component of mixed media. On the one hand, it acts on H2 receptors in the postsynaptic membrane of intrinsic primary afferent neurons, resulting in a long-lasting and slow excitatory postsynaptic potential effect, which enhances the synchronous prolongation of the intestinal wall and secretory activity of mucosal epithelial cells (Nemeth et al., 1984). On the other hand, histamine and prostaglandin can inhibit sympathetic nerve activity, which forms inhibitory synapses with submucosal nerves, and increase the excitability of submucosal secreted motor neurons. After that, submucosal motor neurons release VIP and Ach to promote the secretion of water, electrolytes and mucus in the mucosa (Blandizzi et al., 2000; Liu et al., 2003).

In addition, intramuscular gut neurons themselves also express FcepsilonR1s (FcεRIs) (van der Kleij et al., 2010), which are directly activated by IgE during allergic reactions, leading to muscle contraction and diarrhea symptoms, and the production of histamine to further activate MCs and aggravate food allergic reactions (Yashiro et al., 2021).

## 4 Conclusion

The immune system and nervous system can sense various stimuli inside and outside the body and communicate with each other, helping to maintain host health and homeostasis. Skin, lung, and intestine are the barrier organs of the nervous system and immune cells densely distributed. There are more and more evidences of the existence of neuroimmune units and the close interaction between neuroimmune cells. Elucidating the mechanisms of neuroimmune interaction may have important implications for maintaining and reconstructing tissue homeostasis and discovering new therapeutic targets for barrier organ infectious diseases, inflammatory diseases and allergic diseases.

It is important to note that most of current research methods in the study of neuroimmune drugs promote, physical injury, chemical stimulation, electrical stimulation, etc., while providing preliminary evidence of neuroimmune signal can influence each other, but neuroimmune interaction of group autonomy mechanism is still unclear, and on the study of the neuroimmune cells of the organ is still insufficient. In the future, combined advanced technologies such as tissue-specific optogenetics, chemical genetics and intercellular labeling systems are expected to further explore the cellular intrinsic mechanisms of neuro-immune interactions.
